# Usefulness of D-Dimer and Ultrasonography Screening for Detecting Deep Vein Thrombosis in Patients with Spinal Cord Injury Undergoing Rehabilitation

**DOI:** 10.3390/jcm10040689

**Published:** 2021-02-10

**Authors:** Magdalena Mackiewicz-Milewska, Małgorzata Cisowska-Adamiak, Jerzy Pyskir, Iwona Świątkiewicz

**Affiliations:** 1 Department of Rehabilitation, Nicolaus Copernicus University in Toruń, Collegium Medicum in Bydgoszcz, 85-094 Bydgoszcz, Poland; magmami@onet.eu (M.M.-M.); malgorzata.cisowska@cm.umk.pl (M.C.-A.); 2 Department of Biophysics, Nicolaus Copernicus University in Toruń, Collegium Medicum in Bydgoszcz, 85-094 Bydgoszcz, Poland; jerzy_pyskir@cm.umk.pl; 3 Department of Cardiology and Internal Medicine, Nicolaus Copernicus University in Toruń, Collegium Medicum in Bydgoszcz, 85-094 Bydgoszcz, Poland; 4 Division of Cardiovascular Medicine, University of California San Diego, La Jolla, CA 92037, USA

**Keywords:** spinal cord injury, venous thrombosis, hemostasis, D-dimer, ultrasonography, rehabilitation, plegia, paralysis

## Abstract

Patients with spinal cord injury (SCI) are at an increased risk of deep vein thrombosis (DVT). This study aims at assessing usefulness of D-dimer and compressive Doppler ultrasonography (CDUS) for detecting DVT in patients undergoing rehabilitation at various time-points post-SCI. One-hundred forty-five patients were divided into three groups based on time elapsed since SCI: I (≥3 weeks to 3 months), II (≥3 to 6 months), and III (≥6 months). On admission, D-dimer plasma level measurement and CDUS of the lower limbs venous system were performed. DVT was diagnosed using CDUS in 15 patients (10.3% of entire group), more frequently in group I (22.2% of group) and II (11.7%) compared to group III (1.5%). Most DVT patients received thromboprophylaxis (80%) and were asymptomatic or mildly symptomatic (60%). Median D-dimer was elevated in patients with DVT from all groups, and also patients without DVT from groups I and II, but not group III. D-dimers were higher in patients with DVT than without DVT in the entire group (*p* = 0.001) and group I (*p* = 0.02), but not in groups II and III. The risk of DVT in SCI patients undergoing rehabilitation and thromboprophylaxis including asymptomatic or mildly symptomatic cases, is high within 6 months post-injury, and especially within 3 months. Measurement of D-dimer level should be complemented by routine CDUS for detecting DVT within 6 months post-SCI. Over 6 months, the usefulness of D-dimer screening alone is better for DVT detection.

## 1. Introduction

Venous thromboembolism (VTE), especially deep vein thrombosis (DVT), is a serious complication of spinal cord injury (SCI) that can be a potential life-threatening condition, especially in patients with accompanying para- and tetraparesis [1,2,3,4,5]. VTE is mainly observed in the acute period after trauma, although VTE can also occur in the chronic phase post-injury, albeit less frequently [1,2,3,4]. According to Godat et al. [1], the VTE risk, although it decreases with time from the injury, never goes back to baseline level. The risk of DVT and pulmonary embolism (PE) in patients with SCI is 16.9 and 3.6 times higher for a period of up to 3 months after injury than in the general population [6]. The incidence of DVT ranges from 4.8 to 100% of patients after SCI who do not receive VTE prophylaxis [7,8,9], while DVT and PE were observed in 1.8 to 45% and 3 to 6% of patients undergoing thromboprophylaxis, respectively [10]. In addition, asymptomatic DVT occurs frequently in patients with SCI, e.g., clinical evidence of DVT was present in as low as 20% of SCI rehabilitated patients with confirmed DVT [11,12,13,14].

Screening tests for the detection of DVT include measuring D-dimer plasma level and performing ultrasound examination of the limb(s) venous system using compression and Doppler study (CDUS) [5,15,16]. Ultrasound examination has become the primary noninvasive diagnostic method for DVT diagnosis, which has a good sensitivity and specificity compared to other screening tests [5,15,16]. D-Dimer testing plays an important role in ruling out DVT; however, a usefulness of D-dimer screening for DVT detection in SCI patients at various time-points after injury has not been well established [15]. D-dimer testing and CDUS are carried out in patients with SCI undergoing rehabilitation during acute, subacute and late period post-injury, although routine DVT screening is not recommended in asymptomatic SCI patients receiving thromboprophylaxis [15,16]. Despite the rationale for screening DVT in SCI patients, the existing evidence does not support making a recommendation for or against the use of screening tests for DVT in SCI patients undergoing rehabilitation and receiving VTE prophylaxis [15,16]. Because of the lack of established guidelines for DVT screening in SCI patients and our observations of frequent occurrence of both asymptomatic DVT and non-specific edema of the lower limb(s) in the absence of DVT in patients with SCI undergoing rehabilitation, we find it important to address this topic.

This study aims at assessing usefulness of measuring D-dimer plasma level and performing CDUS for DVT detection in SCI patients undergoing rehabilitation at various time-points post-SCI in the absence of such screening in the acute period after injury.

## 2. Material and Methods

### 2.1. Study Design

This prospective study included patients with SCI who were hospitalized for the rehabilitation at the Department of Rehabilitation, the University Hospital No. 1 in Bydgoszcz, Poland, from 2007 to 2018. The study population involved consecutively admitted patients who were directly transferred to our center from the acute phase treatment department for the early rehabilitation phase along with others participating in the late rehabilitation course, several months or even years after the injury. Every patient with SCI admitted to the Department of Rehabilitation in the period from 2007 to 2018 who agreed to participate and had no contraindications to be transferred to another department for CDUS was included in the study. History of VTE and current antithrombotic treatment were not the reasons for exclusion from the study.

Based on previous studies on the time course of the occurrence of VTE events post-SCI [2,3,4], the patients were divided into the following three groups according to time elapsed since injury: group I (≥3 weeks to 3 months, subacute phase), group II (≥3 months to 6 months, early chronic phase), and group III (≥6 months, late chronic phase). The American Spinal Injury Association Impairment Scale (AIS), time from injury, type of paresis, cause of the injury, presence of heterotopic ossifications, pressure ulcers, urinary tract infections, and use of thromboprophylaxis were assessed upon admission to the hospital for the rehabilitation. Following guidelines on antithrombotic therapy for VTE disease, routine thromboprophylaxis with low-molecular-weight heparin (LMWH) was administered to patients with acute SCI unless it was contraindicated because of high bleeding risk early after injury [16,17,18]. LMWH was initiated in the acute treatment center before admission to our center and was continued for up to 3 months. For patients who were undergoing rehabilitation after acute SCI and for preventing VTE recurrence, the continuation of thromboprophylaxis was recommended. All patients underwent clinical examination for an assessment of paresis as well as clinical symptoms of DVT such as edema, pain, and redness of the lower limbs. Due to frequent sensory disturbances and/or disordered pain perception in SCI patients, we assumed that the main clinical symptoms of DVT include sudden edema and redness of the limb(s). So, the final analysis of clinical symptoms did not include an assessment of pain. Measurements of D-dimer plasma level and platelet count and performing CDUS for detecting deep or superficial vein thrombosis of the lower limbs were done in the first week of hospitalization.

The study was conducted in accordance with the Declaration of Helsinki. Approval from the Bioethics Committee of the Nicolaus Copernicus University in Toruń, Collegium Medicum in Bydgoszcz, Poland was obtained (KB 515/2006 and KB 295/2011). All patients provided informed consent.

### 2.2. Ultrasound Examination of the Lower Limbs Venous System

The CDUS of the lower limbs venous system was performed by an experienced examiner in the Department of Radiology, the University Hospital No. 1 in Bydgoszcz, Poland. The ultrasound examiner was blinded to the results of the D-dimer testing. Standard protocol of CDUS consisted of examination of superficial and deep veins of the lower limbs from the groin to the ankle. The examination consisted of evaluation of an intravascular thrombus presence, a compression B-scan, and a color Doppler flow imaging. A normal study was characterized by an absence of visible intravascular echoes indicating a thrombus, complete venous compressibility, and a Doppler velocity signal that was spontaneous and phasic. An abnormal study was characterized by a dilated incompressible vessel, with the presence of low-level intravascular echoes indicating a thrombus and an absent or continuous Doppler velocity signal.

### 2.3. D-dimer Level and Platelet Count Measurement

Plasma levels of D-dimer were determined by INNOVANCE^®^ D-Dimer (Siemens Healthcare Diagnostic Products GmbH, Erlangen, Germany), which is a particle-enhanced immunoturbidimetric assay for the quantitative determination of cross-linked fibrin degradation products (D-dimers). Platelets were counted with a Sysmex hematology analyzer. D-dimer plasma concentration and platelet count were considered within normal reference ranges when D-dimer was ≤500 ng/mL and platelet count was between 150 and 450 G/L (where G refers to 10^9^).

### 2.4. Statistical Analysis

Statistical analyses were carried out using the Statistica 13.1 software (StatSoft Europe GmbH, Hamburg, Germany). Before performing the statistical comparisons of various variables between groups of patients with DVT and without DVT, the normality of the distribution of variables was assessed using the Shapiro-Wilk test. Normally distributed continuous variables were compared with the Chi-squared test, while those variables not following a normal distribution were analyzed using the Mann-Whitney U test. The differences between groups I, II, and III were analyzed using the Kruskal–Wallis test. Statistical significance was assumed at the level of *p* < 0.05. The median and quartile values (lower and upper) or the mean and standard deviation values were used to describe the variables.

We performed a post-hoc power analysis, which indicated that our study has a sufficient statistical power to detect significant differences in D-dimer concentrations between SCI patients with and without DVT. Specifically, using the GPower software (v.3.1.9.7) [19], we calculated that the sample size of *n* = 145 consisting of 15 patients from DVT group and 130 patients from non-DVT group provides 89% power at the 0.05 significance level to detect an effect size of 0.88 for the D-dimer difference between DVT and non-DVT groups observed in our study. We note that this effect size is highly consistent with previously observed effect size of 0.86 in the population of patients after SCI in the study of Cho et al. [20].

## 3. Results

Initially, the study involved 149 SCI patients hospitalized from 2007 to 2018 at our center for participating in the rehabilitation program. Four patients were excluded from the study because of serious respiratory disorders and infections requiring isolation, which precluded their transfer to the Department of Radiology for performing CDUS. Ultimately, 145 patients were enrolled in the study. The clinical characteristics of whole study group are given in Table 1.

The majority of patients in the entire study group were men (79% of total) aged 37.1 ± 15.1 years old vs. 38 ± 18 years for women. The mean time elapsed since injury was 14.2 ± 26.2 months. The cervical spine injury, tetraparesis, AIS A, and urinary tract infections were diagnosed in almost half of the whole study group. Nine patients (6% of total) had a history of VTE including six patients who had VTE event after SCI (but before admission to our center) despite thromboprophylaxis. LMWH was administered for VTE prophylaxis in the majority of patients (58% of total). The PE occurred during the hospitalization for rehabilitation in one patient (0.7% of total). The median D-dimer level in the entire study group was elevated (> 500 ng/mL), while the platelet count was within a normal range.

There were 45 patients in group I (31% of total), 34 in group II (23%), and 66 in group III (46%). The age of patients from groups I, II, and III was 42.3 ± 17.2 years, 33.5 ± 15.5 years, and 35.8 ± 13.8 years, respectively (*p* = 0.05 for group I vs. II, *p* = 0.189 for group I vs. III, and *p* = 1.0 for group II vs. III). The mean time elapsed since SCI was 1.5 ± 0.5 months, 4.1 ± 0.75 months, and 28 ± 34.1 months in groups I, II, and III, respectively (*p* = 0.0001 for group I vs. II; *p* < 0.0001 for group I vs. III; *p* < 0.0001 for group II vs. III). LMWH was used more frequently in group I (91.1% of patients) compared to group II (76.5%) and group III (25.7%) (*p* = 0.798 for group I vs. II, *p* < 0.0001 for group I vs. III, and *p* = 0.0001 for group II vs. III). It is clinically justifiable that LMWH is administered most frequently for VTE prophylaxis and treatment in patients with most recent SCI and higher thromboembolic risk.

In groups I and II, the median D-dimer level exceeded the laboratory norm and was 2235 (1140; 3920) ng/mL and 769.5 (319;1201) ng/mL, respectively (*p* = 0.001 for group I vs. II). In group III, the median D-dimer was lower compared to groups I and II, and was within a normal range, i.e., it was 299 (203;700) ng/mL (*p <* 0.0001 for group I vs. III; *p* = 0.05 for group II vs. III). In groups I, II, and III, the median platelet count was within normal range, i.e., platelet counts were 265 (230;357) G/L; 253.5 (238;335) G/L; and 233 (194;277) G/L, respectively (*p* = 1.0 for group I vs. II; *p* = 0.038 for group II vs. III; *p* = 0.011 for group I vs. III).

In the entire study group, DVT was diagnosed using CDUS in 15 patients (10.3% of total) (Table 1). The patients with DVT and patients without DVT are denoted as DVT group (*n* = 15 patients) and non-DVT group (*n* = 130 patients). The clinical characteristics of specific patients with DVT are depicted in Table 2. The proximal, distal, and both proximal and distal DVT were diagnosed with CDUS in seven (47% of DVT patients), four (27%), and four (27%) patients, respectively. Bilateral DVT was found in two patients (13% of all DVT patients), whereas 13 patients (87%) had unilateral DVT.

The clinical characteristics of the DVT group and the non-DVT group in the entire study population are shown in Table 1. No significant differences were found between the DVT and non-DVT groups for age and gender. The time elapsed since injury was significantly shorter in the DVT group compared to the non-DVT group (4.5 ± 10 vs. 15.4 ± 27.4 months, *p* = 0004). LWMH was administered more frequently to patients with DVT compared to those without DVT; however, the difference was not statistically significant (80% vs. 55% of patients, *p <* 0.05). The median D-dimer level was significantly higher in DVT group compared to non-DVT group (2875 ng/mL vs. 565 ng/mL, *p* = 0.001). No significant differences were found between groups DVT and non-DVT for other parameters (Table 1). However, patients with DVT were likely to be older and have more frequent urinary tract infections, flaccid paresis of the lower limbs, lumbosacral segment injury, and AIS C. Most patients with the diagnosis of post-thrombotic syndrome from non-DVT group were over six months post SCI, had a known history of VTE, and were on thromboprophylaxis.

DVT occurred more frequently in group I (10 patients, 22.2% of the group) and group II (4 patients, 11.7%) compared to group III (1 patient, 1.5%) (*p* < 0.05 for group I vs. II; *p* = 0.02 for group I vs. III; *p* = 0.0002 for group II vs. III). The clinical characteristics of patients with DVT (DVT groups) and without DVT (non-DVT groups) in groups I, II, and III are shown in Table 3. The comparisons between patients with and without DVT were made for groups I and II. Such a comparison was not possible for group III given that there was only one patient with DVT in group III.

In groups I and II, no significant differences in age and gender were found between DVT and non-DVT groups. The patients with DVT in group I were likely to have shorter time elapsed since SCI compared to patients without DVT, while the time since injury was significantly shorter in DVT patients from group I compared to DVT patients from group II. In group I, 80% of DVT patients received LMWH compared to 94% of patients in whom DVT was not detected with CDUS. In group II, LMWH was administered to all patients from DVT group vs. 73% of patients without DVT.

Median D-dimer levels were elevated in patients with DVT from all groups (i.e., group I, II, and III) and in patients without DVT from group I and II (Table 3). In group I, the median D-dimer level was significantly higher in patients with DVT compared to patients without DVT (*p* = 0.02) (Table 3). In group II, no significant difference in D-dimer was observed between the DVT group and non-DVT group. Patients with DVT from group I had higher median D-dimer concentration compared to patients with DVT from group II (*p* = 0.009). The platelet counts did not differ significantly between DVT and non-DVT patients in both groups I and II (Table 3).

No significant differences were found between patients with and without DVT in groups I and II for other parameters except for the post-thrombotic syndrome in group I, which was more frequent in patients with DVT than without (Table 3).

None of the 15 patients with DVT had all clinical symptoms of DVT. Of the DVT group, six patients (40% of total) had both edema and redness of the lower limb(s), six patients (40%) had only limb(s) edema, and three patients (20%) had no symptoms of DVT. No symptoms or only limb(s) edema was observed in 20% and 40% of patients in group I, 0% and 50% in group II, and 100% (one patient) and 0% in group III, respectively. Of 130 patients in whom DVT was not detected using CDUS, 61 patients (47% of non-DVT group) had swelling or edema of the lower limbs, mainly around the ankles.

## 4. Discussion

This study provides evidence that the risk of DVT in patients with SCI undergoing rehabilitation within subacute and early chronic period post-injury is high despite VTE prophylaxis. In addition, DVT was asymptomatic or mildly symptomatic in the majority of rehabilitated SCI patients. The findings of this study support the recommendation for the routine use of screening tests for DVT detection in SCI patients undergoing rehabilitation within 6 months post-injury despite receiving VTE prophylaxis. Importantly, D-dimer testing alone seems to be insufficient and CDUS is needed in support of comprehensive and safe rehabilitation of SCI patients, especially in the subacute and early chronic period after injury.

The primary findings of our study are: (i) DVT was diagnosed using CDUS in ~10% of patients with SCI undergoing rehabilitation, (ii) DVT was the most frequent within the first 3 months post-injury (~22% of patients) compared to the period of 3 to 6 months (~12%) and over 6 months (~2%), (iii) 80% of patients with diagnosed DVT received thromboprophylaxis, (iv) none of DVT patients had all clinical symptoms of DVT, while 60% of patients were asymptomatic or mildly symptomatic, (v) median D-dimer was elevated (>500 ng/mL) in patients with DVT regardless of the time since injury and in patients without DVT within 6 months post-SCI, (vi) while D-dimer levels were significantly higher in patients with DVT than without DVT within 3 months post-SCI, no differences were observed between patients with and without DVT over 3 months after injury.

The main rationale for pursuing this study on DVT screening in SCI patients stems from our long-term observation of asymptomatic or mildly symptomatic DVT and frequent occurrence of non-specific lower limb(s) edema in SCI patients without thrombotic changes in ultrasound examination. It should be emphasized that the typical triad of the DVT symptoms of the lower limb(s)—edema, pain and redness—was not observed in any of the patients in our study, most likely due to the presence of sensory disturbances in SCI patients, which may impair or abolish pain perception.

DVT is a common and serious complication of SCI, which if untreated can result in PE and death. The greatest risk of DVT occurs in the acute period after SCI and remains high up to 3 months after the injury [1,6,21,22]. After 1 year, the DVT risk goes down to 2.1% and after 2 years to 1%, which is still higher compared to the general population [1,21,22]. In a retrospective study of a group of 47,916 patients after SCI, Chung et al. [6] found that the risk of developing DVT in the first 3 months was 16 times higher than in the general population. This is consistent with our results in which the risk of DVT was greatest in the early rehabilitation group up to 3 months after the injury where 22.2% of patients developed DVT. In the 3 to 6-month group, 11.7% of patients had DVT, while only 1.5% were diagnosed within the group more than 6 months after injury. Our diagnosis of DVT using ultrasound screening in patients over 3 months after the injury is similar to 7.9% reported in our previous study [4]. In the 2016 study, which included 63 patients over 3 months after SCI, we found five cases of DVT, four of them within 6 months after SCI and one in later years [4]. In the present study, we examined 95 patients over 3 months after the injury, and we did not observe an increased number of DVT cases compared to the previous study.

The detectability of DVT after SCI in the early post-trauma period increases with ultrasound screening. Aito et al. [11] showed that when diagnosis was based on clinical symptoms and venography, the incidence of acute DVT was 9% in a group of 362 patients; however, by using ultrasound examination, the diagnosis rate increased to 27% in a group of 130 patients. The incidence of PE in the first group was 4.3%, that was fatal in 3.5%. In the second group the incidence of PE was 0.77% (1 patient) with no fatal case. Another study by the same author [11] showed similar results, i.e., 26 % of patients who had a routine ultrasound exam were diagnosed with DVT; but these patients had delayed initiation of thrombotic prevention. Only 2% of patients from this study had DVT detected by ultrasound if thromboprophylaxis was started within 72 h after the injury.

Detection of DVT by ultrasound screening in the early post-SCI period has been studied by several investigators. Ultrasound screening for DVT in the lower extremity venous system made two to 21 days after trauma resulted in diagnosis of cases in 21 to 45% of patients [10,23]. Agarwal et al. [8] studied a group of rehabilitated patients after SCI for an average of eight days after injury by ultrasound scanning. He diagnosed DVT in 1.8% of patients receiving thrombosis prophylaxis and 3% without prophylaxis and found that fever and calf edema correlated well with DVT occurrence. Do et al. [12] investigated 185 patients admitted for rehabilitation after SCI without pharmacological thromboprophylaxis who underwent a Doppler ultrasound examination and found DVT in 27.6% of patients. Among patients up to 1 month after injury, DVT was diagnosed in 26.8%, while in the group of 1 to 3 months after injury, DVT was diagnosed in 32.1%. Sachdev et al. [24] tested 71 patients rehabilitated after SCI by ultrasound exam, and in spite of receiving pharmacological antithrombotic prophylaxis, 24 patients (33.8%) were diagnosed with DVT. Yelnik et al. [25] identified DVT in 27.8% of 147 recent SCI patients admitted to rehabilitation. Similar results were obtained by Cho et al. [20] in a study of 45 patients rehabilitated five to 90 days after injury without thromboprophylaxis. DVT was detected by screening test in 14 patients (31% of total) in which the presence of DVT correlated with higher D-dimer values. In the present study, we diagnosed DVT in ~22% of patients up to 3 months post-SCI despite the fact that 80% of them received the thromboprophylaxis with LMWH.

Because DVT in post-SCI patients is often asymptomatic or mildly symptomatic, testing for DVT in patients undergoing rehabilitation is especially important to conduct safe rehabilitation and prevent severe life-threatening complications such as PE. The high risk of developing asymptomatic DVT with an increased rate of DVT recurrence implies a need of extended anticoagulant therapy for primary treatment, which in SCI patients should be continued for at least 3 to 6 months [26]. Moreover, because SCI patients had persistent chronic risk factors for DVT and PE, the American Society of Hematology 2020 guideline panel suggests indefinite antithrombotic therapy for secondary prevention after completion of the primary treatment rather than stopping anticoagulation [26]. In our present study, six patients diagnosed with DVT had edema and redness of the limbs (40% of DVT patients), while three patients (20%) showed no clinical symptoms, and in the remaining six patients (40%) only slight edema around the ankles was observed. It should be noted that in 61 patients (47%) without DVT detected using CDUS, non-specific edema of the lower limbs, especially around the ankles, were observed without the features of DVT in CDUS.

Several investigators have reported the occurrence of asymptomatic DVT in patients with SCI, but the data on the incidence of silent thrombosis varies widely according to different studies. Wada et al. [13] postulated one cause of asymptomatic DVT in patients after SCI as a disturbance in the functioning of the autonomic system, which reduced the response to vasculitis and subsequent lower limb edema. Do et al. [12] found that out of 51 SCI patients diagnosed with DVT by ultrasound screening, only 10 (~20%) were symptomatic. Most of these patients (67%) had isolated distal thrombosis, while 27% had proximal thrombosis. It is interesting that Halim et al. [14] detected asymptomatic thrombosis in 75% of patients without antithrombotic prophylaxis, while in the group with thromboprophylaxis all DVTs were symptomatic. Aito et al. [11] and Kulkarni et al. [27] found that 65% of patients after SCI were asymptomatic for DVT, while the percentage increased to 83% in those patients receiving antithrombotic prophylaxis.

D-dimer testing in patients with clinical suspicion of DVT is widely used; however, the sensitivity of D-dimer testing for the diagnosis of DVT after SCI has been shown to be 78%, and its specificity is 40%, with a negative predictive value of 92% [28]. In patients after SCI, especially during the early post-trauma period, the level of D-dimer is usually high in patients both with DVT and without [29]. In the present study, the median level of D-dimer exceeded the normal range in the entire study group and in patients within 6 months since SCI. It remained high in patients with DVT regardless of the time elapsed since injury and in patients without DVT up to 6 months after SCI. The values for D-dimer in patients with DVT compared to those in the non-DVT group were significantly higher in patients within 3 months post-SCI. The median level of D-dimer also exceeded the norm in group of patients 3 to 6 months after injury, but the values in DVT and non-DVT patients did not differ statistically and were significantly lower than in the group of patients below 3 months post-SCI, especially in patients with DVT. This may indicate that DVT in group II of patients (3 to 6 months after SCI) developed in the acute period post-injury but was not diagnosed earlier because ultrasound screening was not done and DVT was not fully symptomatic. Our present findings are consistent with our previous study which showed that the level of D-dimer remained high in patients up to 3 months after SCI, with the median of 2540 ng/mL and average value of 4488 ng/mL [30]. Matsumoto et al. [5] observed very high average D-dimer values of 14,600 ng/mL among patients several days after SCI, which did not differ significantly between groups with and without DVT.

A need for performing screening tests to detect DVT in patients undergoing rehabilitation after SCI is not well established. Based on our findings, screening with D-dimer testing alone for DVT detection is insufficient, and it is necessary to perform CDUS to diagnose DVT regardless of using thromboprophylaxis or presence of clinical symptoms of DVT, especially in patients up to 6 months post-injury. This conclusion is consistent with findings of Kumagai et al. [31], who showed that ultrasound examination combined with D-dimer screening identified more asymptomatic DVT than D-dimer screening alone. Importantly, the American College of Chest Physicians evidence-based clinical practice guidelines on the diagnosis of DVT recommend performing a compressive ultrasonography in patients with a high pretest probability of lower extremity DVT, while indicating that D-dimer assays should not be used as stand-alone tests to rule out DVT in this population of patients [32]. A high pretest probability of DVT can refer to the population of patients with SCI, which is characterized by the presence of chronic (persistent) risk factors for the development of VTE [16,18,26]. While there is some disagreement on this aspect, we note that Furlan et al. [15] indicated that there is insufficient evidence to support (or refute) a recommendation for routine screening for DVT in asymptomatic patients with acute traumatic SCI under thromboprophylaxis. However, Furlan et al. [15] also recognized that screening could detect asymptomatic DVT in 22.7% of these individuals and weekly DVT screening during the first 13 weeks post-SCI could be useful for detection of most asymptomatic DVT events in such population. They also pointed out that D-Dimer, ultrasound, and MR venography can serve as potentially useful screening tests for DVT in the SCI population in future research studies.

The high risk of VTE complications in SCI patients is associated with several factors including venous stasis resulting from immobilization, paresis or paralysis, decreased muscle contractility, as well as previous VTE, older age, infections, and hemostasis disorders [2,4,6,21,29,33]. In our study, the risk of DVT appeared to be higher in SCI patients at older age as well as those with flaccid paresis of the lower limbs, AIS C, lumbosacral segment injury, and urinary tract infections. Similar results were obtained by Green et al. [34] who reported that flaccid paresis, but not spastic paresis, was an independent risk factor for DVT. In addition, Do et al. [12] showed that the absence of spasticity was an independent risk factor for the development of DVT in patients after SCI. Although half of DVT patients in our study had complete functional impairment, patients with AIS C and less severe paresis also seemed to have a higher risk of developing DVT. Masuda et al. [33] also showed that almost all of the DVT patients demonstrated severe paralysis classified as AIS C or greater and the highest rate of DVT was observed among patients with AIS C paralysis. Venous blood congestion due to paralysis in the lower limbs can predispose patients to thrombogenesis and VTE complications [6,21,33]. While the significance of other risk factors, such as Virchow triad, is beyond any doubt especially significant in the acute or subacute period post SCI, chronic immobility in patients with SCI due to paralysis is considered as a significant chronic (persistent) risk factor for the development of VTE [16,18,26,32,35,36]. However, Agarwal et al. [8] concluded that the level of injury and the AIS grade were not statistically related to the occurrence of DVT. Urinary tract infections, which are the most common infections in patients with both acute and chronic SCI, were likely to be more frequent in patients with DVT in our study. Smeeth et al. [37] also reported an increased risk of VTE in patients with urinary tract infection. It has also been shown that older age is associated with increased risk of VTE in the general population and patients with SCI, which is consistent with our findings [1,6,36]. A higher incidence rate of VTE with increasing age can result from various risk factors for thrombosis associated with advancing age such as deterioration of cardiopulmonary systems and greater incidence of comorbidities [6].

The high risk of VTE after acute SCI justifies the routine use of pharmacologic thromboprophylaxis, which should be initiated as early as possible within the limits of safety and feasibility [16,17,18,26,38]. Thromboprophylaxis with LMWH is effective in SCI patients and maintains relatively low risk of bleeding complications [16,17,18,26]. However, the effectiveness of thromboprophylaxis may be limited in high-risk populations due to the combination of a very high thrombogenic stimulus in patients with acute SCI and the delay between the injury and the start of prophylaxis [11,25,38]. Our findings indicate that pharmacologic thromboprophylaxis administered in the chronic phase of SCI to patients with post-thrombotic syndrome may decrease a risk of DVT.

### Study Limitations

Screening for detecting DVT was performed only once during the first week of hospitalization for the rehabilitation program. During further course of hospitalization, CDUS for DVT detection was performed only in patients who developed clinical symptoms of DVT of the lower limb(s). It is possible that during the rehabilitation period, which lasted several weeks to several months, new asymptomatic DVT could appear which was not diagnosed due to the lack of clinical symptoms and routine periodic ultrasound screening.

## 5. Conclusions

The risk of DVT in SCI patients undergoing rehabilitation and thromboprophylaxis is high within 6 months post-injury, and especially within 3 months. Frequent occurrence of asymptomatic or mildly symptomatic DVT reinforces a need for DVT screening. Testing for the D-dimer level for DVT detection in SCI patients undergoing rehabilitation within 6 months post-injury is helpful but does not allow accurate DVT diagnosis due to high D-dimer levels in SCI patients without DVT. Measurement of D-dimer level should be complemented by routine CDUS for detecting DVT within 6 months post-SCI. Over 6 months, the usefulness of D-dimer screening alone is better for DVT detection. As DVT risk significantly decreases over 6 months after SCI, the usefulness of routine ultrasound screening in this period is debatable due to the low number of DVT cases detected.

D-dimer testing alone seems to be insufficient and CDUS is needed in support of comprehensive and safe rehabilitation of SCI patients, especially within 6 months after injury. Further studies on usefulness of D-dimer and CDUS screening for DVT detection are needed for a larger number of SCI patients, especially those undergoing rehabilitation within the period of 3–6 months post-injury.

## Figures and Tables

**Table 1:** Characteristics of whole study group and groups of patients with deep vein thrombosis (DVT group) and without deep vein thrombosis (non-DVT group).

Parameter	Whole Study Group (*n* = 145)	DVT Group (*n* = 15)	Non-DVT Group (*n* = 130)	*p*-Value
Age (years)	37.3 (±15.7)	41.4 (±15.9)	36.7 (±15.6)	0.257
Gender: female/male (*n*, %)	30/115 (21/79)	3/12 (20/80)	27/103 (21/79)	0.46
D-dimer (ng/mL)	700 (260;1590)	2875 (1005;7505)	565 (256;1500)	0.001
PLT (G/L)	250 (204;330)	272 (239.5;429.5)	249 (204;320)	0.329
Time since injury (months)	14.2 (±26.2)	4.5 (±10)	15.4 (±27.4)	0.0004
Cervical spine injury *(n*, %)	69 (48)	6 (40)	63 (48)	0.951
Thoracic spine injury (*n*, %)	60 (41)	6 (40)	54 (42)	0.925
Lumbosacral spine injury (*n*, %)	18 (12)	3 (20)	15 (12)	0.316
Tetraparesis (*n*, %)	66 (46)	5 (33)	61 (47)	0.332
Flaccid paraparesis (*n*, %)	30 (21)	4 (27)	26 (20)	0.721
Spastic paraparesis (*n*, %)	49 (34)	6 (40)	43 (33)	0.664
AIS A (*n*, %)	68 (47)	7 (47)	61 (47)	0.938
AIS B (*n*, %)	49 (34)	4 (27)	45 (35)	0.617
AIS C (*n*, %)	28 (19)	4 (27)	24 (18)	0.606
Urinary tract infection (*n*, %)	59 (41)	7 (47)	52 (40)	0.675
Heterotopic ossifications (*n*, %)	23 (16)	0	23 (18)	
Decubitus ulcers (*n*, %)	14 (10)	2 (13)	12 (9)	0.874
Superficial VT of lower limb(s) (*n*, %)	11 (8)	0	11 (9)	
Post-thrombotic syndrome (*n*, %)	12 (8)	3 (20)	9 (7)	0.409
Use of LMWH (*n*, %)	84 (58)	12 (80)	72 (55)	0.119

For most parameters, data represent the number of patients (*n*) including the percentage of total number (in parenthesis). For D-dimer level and platelet count, median values with lower and upper quartiles (in parenthesis) are given. For age and time since injury, mean values with standard deviation (in parenthesis) are given. Legend: DVT group (patients with DVT); non-DVT group (patients without DVT); *p* values are given for the difference between the DVT group and non-DVT group. Abbreviations: AIS—The American Spinal Injury Association Impairment Scale; AIS A—impairment complete according to AIS; AIS B and C—impairment incomplete according to AIS; DVT—deep vein thrombosis; G—10^9^; LMWH—low molecular weight heparin; PLT—platelet count; VT—venous thrombosis.

**Table 2:** Clinical characteristics of specific patients with deep vein thrombosis.

Sex	Age (years)	Time Post-SCI (months)	Level of SCI	Paresis Type and Location	AIS	DVT Location	Urinary Tract Infection	PTS	Cause of SCI	LMWH Use	D- dimer (ng/mL)	DVT Signs
M	80	1.5	LS	FLL	C	PTV R and L	-	+	Dis	+	1317	E, R
F	40	0.5	Th	FLL	B	SV R	-	+	I	+	14,700	E, PE
M	35	1	LS	FLL	C	FV, PV, SV R	-	-	Fa	-	15,700	E, R
M	21	1	C	ST	A	FV, PV, SV L	-	-	TA	+	6900	E, R
F	39	1	LS	FLL	C	SV L	+	-	TA	+	3990	E
M	28	2	C	ST	A	FV, PV, SV R	+	-	TA	+	2850	No
M	56	1	Th	SLL	A	PV R and L, FV R	-	-	Fa	+	8051	No
F	30	48	C	ST	B	SV L	+	-	TA	-	531	No
M	23	4	C	ST	C	PV R	-	-	Fa	+	88	E
M	26	2	Th	SLL	A	FV, PV L	+	-	Fa	-	1010	E, R
M	41	4	Th	SLL	A	PV R	+	-	TA	+	1201	E, R PE
M	55	5	Th	SLL	B	PV, SV L	-	-	IN	+	1000	E, R
M	49	4	Th	SLL	A	PV R	+	-	Fa	+	800	E
M	63	1.5	C	ST	A	SV R	+	+	Fa	+	2900	E
M	36	1	C	SLL	B	FV R	-	-	TA	+	6309	E

Abbreviations: DVT—deep vein thrombosis; F—female; LMWH—low-molecular-weight heparin; M—male; PE—pulmonary embolism; PTS—post-thrombotic syndrome; SCI—spinal cord injury. AIS—The American Spinal Injury Association Impairment Scale; AIS A—Impairment is complete according to AIS; AIS B and C—Impairment is incomplete according to AIS. Cause of SCI: Dis—discopathy; Fa—fall; I—ischemia with myelitis; IN—inflammation of spine; TA—traffic accident. DVT location: FV—femoral vein; PTV—posterior tibial veins; PV—popliteal vein-; SV—sural veins; R—right; L—left. DVT signs: E—limb edema; R—limb redness. Level of SCI: C—cervical; Th—thoracic; LS—lumbosacral. Paresis type and location: SLL—spastic of lower limbs; FLL—flaccid of lower limbs; ST—spastic tetraparesis.

**Table 3:** Characteristics of patients with (DVT group) and without (non-DVT group) deep venous thrombosis in groups I, II, and III.

Parameter	Group I		Group II		Group III		
	DVT (*n* = 10)	Non -DVT (*n* = 35)	DVT (*n* = 4)	Non -DVT (*n* = 30)	DVT (*n* = 1)	Non -DVT (*n* = 65)	
Age (years)	42.3 (±17.5)	42.4 (±17.4)	42 (±13.9)	32.3 (±15.6)	30	35.9 (±14)	
Gender: female/male (*n*, %)	2/8 (20/80)	7/28 (20/80)	0/4 (0/100)	7/23 (23/77)	1/0 (100/0)	13/52 (20/80)	
D-dimer (ng/mL)	6309 ^a,c^ (2850;8158)	1680.5 ^a^ (716;3150)	900 ^c^ (444.9;1100.5)	728.5 (319;1360)	531	298 (203;700)	
PLT (G/L)	357 (242;494)	257 (220;335)	218.3 (158.5;278)	253.5 (238;345)	240	233 (194;277)	
Time since injury (months)	1.2 ^d^ (±0.5)	1.6 (±0.5)	4.3 ^d^ (±0.5)	4.1 (±0.8)	48	27.8 (±34.4)	
Cervical spine injury (*n*, %)	4 (40)	10 (29)	1 (25)	14 (47)	1 (100)	39 (60)	
Thoracic spine injury (*n*, %)	3 (30)	15 (43)	3 (75)	18 (60)	0	21 (32)	
Lumbosacral spine injury (*n*, %)	3 (30)	7 (20)	0	49 (13)	0	4 (6)	
Tetraparesis (*n*, %)	3 (30)	8 (23)	1 (25)	14 (47)	1 (100)	39 (60)	
Flaccid paraparesis (*n*, %)	4 (40)	12 (34)	0	7 (23)	0	7 (11)	
Spastic paraparesis *(n*, %)	3 (30)	15 (43)	3 (75)	9 (30)	0	19 (29)	
AIS A (*n*, %)	5 (50)	15 (43)	2 (50)	16 (53)	0	30 (46)	
AIS B (*n*, %)	2 (20)	13 (37)	1 (25)	10 (33)	1 (100)	22 (34)	
AIS C (*n*, %)	3 (30)	8 (23)	1 (25)	4 (13)	0	12 (18)	
Urinary tract infection (*n*, %)	4 (40)	11 (31)	2 (50)	16 (53)	1 (100)	25 (38)	
Heterotopic ossifications (*n*, %)	0	2 (6)	0	8 (27)	0	13 (20)	
Decubitus ulcers (*n*, %)	1 (10)	4 (11)	1 (25)	7 (23)	0	1 (2)	
Superficial VT of lower limb(s) (*n*, %)	0	8 (23)	0	1 (3)	0	2 (3)	
Post-thrombotic syndrome (*n*, %)	3 (30) ^b^	2 (6) ^b^	0	0	0	7 (11)	
Use of LMWH (*n*, %)	8 (80)	33 (94)	4 (100)	22 (73)	0	17 (26)	

For most parameters, data represent the number of patients (*n*) including the percentage of total number (in parenthesis). For D-dimer level and platelet count, median values with lower and upper quartiles (in parenthesis) are given. For age and time since injury, mean values with standard deviation (in parenthesis) are given. Legend: group I (≥3 weeks to 3 months since SCI); group II (≥3 to 6 months since SCI); group III (≥6 months since SCI); ^a^ *p* = 0.02 for group DVT vs. group non-DVT in group I; ^b^ *p* = 0.04 for group DVT vs. group non-DVT in group I; for the remaining parameters *p >* 0.05 for group DVT vs. group non-DVT within groups I, II, and III; ^c^ *p* = 0.009 for group DVT of group I vs. group DVT of group II; ^d^ *p* = 0.005 for group DVT of group I vs. group DVT of group II. Abbreviations: AIS—The American Spinal Injury Association Impairment Scale; AIS A—impairment complete according to AIS; AIS B and C—impairment incomplete according to AIS; DVT—deep vein thrombosis; G—10^9^; LMWH—low molecular weight heparin; PLT—platelet count; SCI—spine cord injury; VT—venous thrombosis.

## Data Availability

The data presented in this study are available on request from the corresponding author. The data are not publicly available due to privacy restrictions.
